# Supplementary material for: The evolution of multicellular complexity: the role of relatedness and environmental constraints
Source: Proc Biol Sci. 2020 Jul 22;287(1931):20192963. doi: 10.1098/rspb.2019.2963 (PMC7423666; doi:10.1098/rspb.2019.2963)
Supplement: Supplementary Information;Raw data tables [file rspb20192963supp1.docx]

**Response to Reviewers comments**

**Associate Editor:
Board Member: 1
Comments to Author:**
Congratulations! We are pleased to accept your manuscript with minor revisions. This is an interesting and well written paper that uses a phylogenetic context captures large scale trends in evolution of multicellularity.

In your revision please address the comments of reviewer 2 with respect to classification of cell types, as well as reviewers 1's comment on prokaryotes.

As well, before publication we also require data and code deposition for reproducibility. While the authors link to a well documented repository containing the data published in a previous analysis, and the updated data is provided in the supplement, the data deposition guidelines are: "To allow others to verify and build on the work published in Royal Society journals, it is a condition of publication that authors make available the data, code and research materials supporting the results in the article." In order to make the work from this publication reproducible, their expanded categorized data set, inferred phylogeny, and associated analysis code should be shared in a dryad repository for this paper.

*The data, phylogeny and original code are now available on Dryad and we have added the link and doi to the Methods section (lines 285 – 286).*

Some citations are missing in the methods section. The ROTL package was described in Michonneau et al 2016 <https://doi.org/10.1111/2041-210X.12593>, and the OpenTree of Life project lists appropriate citations at <https://tree.opentreeoflife.org/about/open-tree-of-life> (citations to the taxonomy, datastore, and synthesis would be appropriate).

*We have added these missing citations.*

**Minor figure comments:**
The resizing of the axes between panel a and b in figure 1 makes the match between them harder to see. Also, consider using different symbols in addition to different colors in panel b  and Figure 4. to make the figure more comprehensible in B/W and for people with limited color vision.

*We have edited the figures so they are more ‘in line’ and comparable. We accept the comment regarding accessibility with Figure 1b, however have decided to leave the colour as is, because 14 different shapes on a small is likely to be harder to read and lessen the Figures impact.*

Figure 3. Could make use of the extra space in the key to provide more visual clarification about the bar charts, and label the axis values for the bar charts.

*We have added the cell types values to the legend, as this would be too small to add to the figure and still be legible (lines 449 – 450).*

Consider B/W printing and color challenges in phylogeny figure as well - e.g. dividing line between ciliates and red algae is hard to see even with color.

*We have changed the colour of the ciliates so that there is a better distinction between the phylogenetic groups.*

**Reviewer(s)' Comments to Author:

Referee: 1

Comments to the Author(s)**
The authors adequately addressed the referees' concerns in their revision. In particular, they clarified the statistical methods used, which answered my main concerns. As such, I believe the manuscript is suitable for publication in the current form.

As a note, in line 265 the authors refer to "prokaryotes and archaea". I think they mean "bacteria and archaea" (or otherwise simply "prokaryotes") since archaea are prokaryotes also.

*We thank the reviewer for spotting this error. We have changed this to ‘prokaryotes’.*

**Referee: 3

Comments to the Author(s)**
The authors have substantially revised the manuscript. It seems that the statistical analyses reveal a trend that is inherent to the data and not imposed by the analyses, which was a major concern raised in the first round of reviews. However, we still feel that some of the problems associated with the classification of cell types in the dataset were not sufficiently addressed. In particular, we would like to see a bit more discussion on how the different authors of the datasets used in this paper classified cell types across different multicellular lineages, and how this affects the results in this paper. This is important because the classification in itself could have implicit biases in the number of cell types counted, especially because a substantial portion of the data is now fairly old (i.e., Bell 1997), and modern molecular techniques have changed our views of what counts as a cell type. Otherwise, the paper is nice!

*We thank the reviewer for their comments. We have now added a section to the Methods (lines 297 – 303) where we go into more detail about how cell types have been defined. We also acknowledge this in the Discussion section (lines 229 – 232).*
